# The Key Differences between Human Papillomavirus-Positive and -Negative Head and Neck Cancers: Biological and Clinical Implications

**DOI:** 10.3390/cancers13205206

**Published:** 2021-10-17

**Authors:** Steven F. Powell, Lexi Vu, William C. Spanos, Dohun Pyeon

**Affiliations:** 1 Cancer Biology and Immunotherapies Group, Sanford Research, Sioux Falls, SD 57104, USA; steven.powell@sanfordhealth.org; 2 Department of Microbiology and Molecular Genetics, Michigan State University, East Lansing, MI 48824, USA; vulexi@msu.edu

**Keywords:** head and neck cancer, head and neck squamous cell carcinoma, human papillomavirus, treatment, clinical outcome, tumor microenvironment, molecular carcinogenesis, microbiome, surgery, de-escalation strategies, clinical trials

## Abstract

**Simple Summary:**

Head and neck cancer (HNC) is the sixth most common cancer, causing almost half a million deaths worldwide every year. The two subtypes of HNC are distinctly associated with smoking/drinking and/or human papillomavirus (HPV) infection. While the incidence of non-viral HNC is decreasing, HPV-positive HNC incidence has dramatically increased in the last few decades. Accumulating evidence from numerous studies shows molecular and clinical differences in mutations, gene expression regulation, treatment responses, and patient survival rates between HPV-positive and HPV-negative HNC. Here, we discuss the current status of HNC research and clinical approaches and suggest unanswered questions and future directions.

**Abstract:**

Head and neck squamous cell carcinoma (HNSCC) is a unique malignancy associated with two distinct risk factors: exposure to typical carcinogens and infection of human papillomavirus (HPV). HPV encodes the potent oncoproteins E6 and E7, which bypass many important oncogenic processes and result in cancer development. In contrast, HPV-negative HNSCC is developed through multiple mutations in diverse oncogenic driver genes. While the risk factors associated with HPV-positive and HPV-negative HNSCCs are discrete, HNSCC patients still show highly complex molecular signatures, immune infiltrations, and treatment responses even within the same anatomical subtypes. Here, we summarize the current understanding of biological mechanisms, treatment approaches, and clinical outcomes in comparison between HPV-positive and -negative HNSCCs.

## 1. Introduction

Head and neck squamous cell carcinomas (HNSCC) comprise squamous cell carcinoma of the oral cavity, nasal cavity, pharynx (oropharynx and hypopharynx), larynx, and tongue. Combining them all, more than 68,000 cases of HNSCC are annually reported, accounting for ~4% of all cancers in the United States [1]. Unlike most other solid cancers, HNSCCs arise from broad anatomical sites where cell type compositions are largely diverse with high heterogeneity. While smoking and drinking are well known as main risk factors, a significant number of HNSCCs in the oropharynx are also associated with infection of viruses such as human papillomavirus (HPV) [2,3,4].

**Figure 1:**
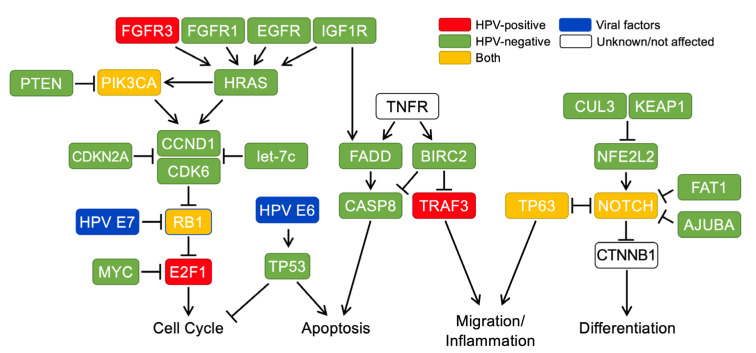
An overview of the key factors and pathways with genome mutations and molecular dysregulations in HPV+ and HPV− HNSCCs [7,10]. Genome mutations and alterations mainly found in HPV+, HPV−, and both HNSCCs are indicated with red, green, and yellow boxes, respectively. The HPV oncogenes E6 and E7 are shown as blue boxes, and unknown or unaffected genes are shown as white boxes. FGFR3, fibroblast growth factor receptor 3; FGFR1, fibroblast growth factor receptor 1; EGFR, epidermal growth factor receptor; IGF1R, insulin like growth factor 1 receptor; PTEN, phosphatase and tensin homolog; PIK3CA, phosphatidylinositol-4,5-bisphosphate 3-kinase catalytic subunit alpha; HRAS, Hras proto-oncogene, GTPase; CCND1, cyclin D1; CDK6, cyclin dependent kinase 6; CDKN2A, cyclin dependent kinase inhibitor 2A; let-7c, microRNA let-7c; RB1, RB transcriptional corepressor 1; MYC, MYC proto-oncogene, BHLH transcription factor; E2F1, E2F transcription factor 1; TP53, tumor protein P53; TNFR, tumor necrosis factor receptor; FADD, fas associated via death domain; CASP8, caspase 8; TRAF3, tumor necrosis factor receptor associated factor 3; CUL3, cullin 3; KEAP1, kelch like ECH associated protein 1; NFE2L2, nuclear factor, erythroid 2 like 2; TP63, tumor protein P63; NOTCH, notch receptor; FAT1, FAT atypical cadherin 1; AJUBA, ajuba LIM protein; CTNNB1, catenin beta 1.

The molecular and clinical differences of HPV-positive (HPV+) and HPV-negative (HPV−) HNSCCs are substantial; and they are now largely considered as two distinct cancers despite their histological similarities [2,5,6]. HPV+ HNSCC is caused by the various oncogenic functions of high-risk HPV E6 and E7, which may efficiently compensate for common oncogenic driver mutations that lead to HNSCC carcinogenesis (Figure 1) [2,5,7]. Indeed, the Cancer Genome Atlas (TCGA) data demonstrate significantly higher mutation loads in HPV− HNSCC than in HPV+ HNSCC [8,9,10].

The incidence of HNSCC in the oropharynx associated with HPV infection has significantly increased in the last few decades [11,12]. The overall incidence of HPV− HNSCC has decreased, probably due to the reduction in smoking populations in the United States [13]. Several recent studies have shown that the oncogenic mechanisms of HNSCCs, particularly between HPV+ and HPV− HNSCCs, are unique and extremely diverse [5,8]. HPV+ HNSCC patients show significantly better survival rates following the standard chemoradiation therapy (CRT) compared to HPV− HNSCC patients [14,15,16]. However, despite these improved outcomes, those with HPV+ HNSCC still develop recurrent/metastatic (R/M) disease as the high-risk subgroup of HPV+ HNSCC. There is little insight to clearly explain the mechanisms that lead to the differential response to CRT and patient survival. Although it is under intense focus, limited tools are available to distinguish those within the high-risk subgroup of HPV+ HNSCC.

This high diversity and heterogeneity of HNSCC make it arduous to identify key targets to develop effective cancer therapeutics and treatment strategies. As a brainstorming effort to overcome these hurdles, here we summarize the current state of our understanding and clinical observations to inform future directions in HNSCC research.

## 2. Molecular Carcinogenesis Is Distinct between HPV+ and HPV− HNSCCs

The genetic landscape of HNSCC carcinogenesis shows two distinct oncogenic pathways, driven by either chemical carcinogens or HPV infection [2]. Smoking and alcohol use have long been characterized as the major risk factors for developing HNSCC, independent of HPV infection [17,18,19]. The carcinogenic nitrosamines formed from tobacco and acetaldehyde from alcohol can be metabolically activated and covalently bound to DNA, forming DNA adducts that disrupt the DNA double helix [20,21]. Persistent DNA adducts lead to hypermutations and chromosomal instability, some of which accidentally dysregulate crucial cellular mechanisms and pathways in cell homeostasis, resulting in cancer progression [22,23]. This effect may be exacerbated by alcohol, as it can act as a solvent to introduce tobacco-related carcinogens into the mucosa of the head and neck region [24,25]. Furthermore, alcohol induces the enzyme encoded by cytochrome p450 2E1 (*CYP2E1*), an activator of nitrosamine metabolism [26]. As a result, alcohol and tobacco act synergistically to promote HNSCC carcinogenesis [19,27]. Beyond alcohol and tobacco, other topical exposures, such as betel nuts, are known to be linked to HNSCC by generating nitroso-derivatives similar to tobacco smoking from alkaloid ingredients [28,29].

In many cases, tobacco is sufficient to exert carcinogenic effects independent of alcohol use. The risk of the development of HNSCC with tobacco alone is higher than alcohol alone. However, the combination of alcohol and tobacco has a multiplicative associated risk [30]. Studies have shown that the reactive oxygen and nitrogen species found in cigarette smoke (CS) are known to lead to oxidative stress and activation of proinflammatory pathways in lung fibroblasts [31,32,33]. Recently, it was shown that fibroblasts exposed to CS have the potential to alter the tumor microenvironment (TME) in HNSCC through similar mechanisms as the lung fibroblasts [34,35]. CS induced oxidative stress and expression of the hypoxia responsive gene monocarboxylate transporter 4 (MCT4) in fibroblasts in vitro [36]. Tumors generated from co-injection with CS-exposed fibroblasts showed a marked increase in the macrophage markers CD45 and CD68 in vivo [37]. CS is also well known to induce immune suppression by modulating various immune mechanisms in other cancers [38,39,40]. Notably, increased macrophages in the TME are a characteristic of HNSCC associated with smoking and immune suppression [37,41,42]. This suggests that smoking dysregulates antitumor immune mechanisms that are distinct from virus-mediated immune suppression [43]. The differential immune dysregulations are further discussed below.

An emerging mechanism of tobacco-induced carcinogenesis is through altered microRNA (miRNA) expression [44,45]. miRNAs are small, non-coding RNAs that play a role in posttranscriptional gene regulation and translation inhibition [46]. Disruption of miRNA regulation by tobacco exposure may lead to changes in major cellular signaling pathways and metabolic processes. Indeed, increased production of nicotine-derived nitrosamine ketone (NNK), a carcinogenic component of tobacco, in HNSCC cells upregulates expression of several oncogenic miRNAs, including miR-21, miR-155, and miR-944, while downregulating expression of the tumor suppressor miRNA miR-422a in HNSCC cells in in vitro culture [47,48]. The upregulation of miR-944 expression in HNSCC induces the secretion of proinflammatory cytokines and activates signal transducer and activator of transcription 3 (STAT3), contributing to tumorigenesis [48]. Using mouse and rat models of tobacco-associated HNSCC, a recent study has shown that the family of miR-30 is significantly downregulated in tumors compared to normal tongue tissue [49]. The cellular targets of the miR-30 family of microRNAs (miRNAs) include messenger RNAs (mRNAs) involved in cell proliferation, differentiation, adhesion, and invasion [50,51]. This downregulation of the miR-30 family has also been observed in the analyses of HNSCC cell lines and TCGA HNSCC datasets [49,52]. However, miRNA targets are context-dependent, many are still unknown including the contributions of HPV, and cell lines and animal models may differ from patient samples. Thus, further studies are needed to establish a causal link between tobacco induced HNSCC development and miRNA alterations.

Conversely, the genetic alterations observed in HPV-driven HNSCCs are predominantly caused by the viral oncogenes E6 and E7 following persistent infection of high-risk HPV, such as HPV16 and HPV18. The HPV genome is also frequently integrated into the host chromosome, occasionally forming concatemers of multiple HPV E6/E7 segments [53,54]. The linearization of the HPV genome creates a breakpoint in the early gene E2, the only transcription factor encoded by the HPV genome [55,56]. While HPV E2 functions as a negative regulator of early viral gene expression, including E6 and E7, integration of the HPV genome frequently truncates E2, preventing transcriptional repression of the E6 and E7 oncogenes [57,58]. High-risk HPV E6 and E7 dysregulate various cellular pathways, including inactivation of the tumor suppressors p53 and pRB, respectively [59]. Furthermore, the varying integration sites have been shown to alter gene expression to promote cancer progression. For example, it has been found that HPV DNA insertion into the RAD51 homolog 2 (*RAD51B*) gene, which is a core component of the DNA double-strand break repair, promotes the formation of alternative transcripts, generating a non-functional RAD51 protein [58,60]. In addition to the E6/E7, a recent study suggests an alternative oncogenic mechanism of the E2/E4/E5 subtype of HPV+ HNSCC containing episomal HPV, which shows fibroblast growth factor receptor (FGFR) activation and p53-dependent cell proliferation [61].

The previous TCGA studies have revealed that HPV+ HNSCC shows significantly lower rates of allelic loss and genetic mutations compared to HPV− HNSCC [8,9]. The coding sequences of several tumor suppressors in HPV− HNSCC contain driver mutations that are not observed in HPV+ HNSCC (Figure 1). One of the most striking contrasts is p53, which is mutated in the vast majority of HPV− HNSCC, but rarely in HPV+ HNSCC [8,62]. In addition, a comparison of HPV+ and HPV− tumor samples from patients of a similar age and tumor site using microarray-based comparative genomic hybridization (maCGH) showed a significantly higher number of chromosomal alterations in HPV− tumors compared to HPV+ tumors [63,64]. Four chromosomal regions, the smallest of which spanned four megabases, were found to be significantly altered in HPV− tumors, whereas no change or a change in the opposite direction were found in HPV+ tumors [63]. In contrast, distinct chromosome aberrations are associated with viral integration sites in HPV+ HNSCC, contributing to cancer progression [65,66]. Taken together, it is likely that expression of the viral oncogenes E6 and E7 is sufficient to inactivate many of the critical tumor suppressor pathways, promoting carcinogenesis without generating somatic mutations in tumor suppressor genes.

In addition to the dysregulation of the common oncogenic pathways, there are gene expression distinctions between HPV+ and HPV− HNSCCs. Our previous studies of global gene expression analysis revealed that expression of cell cycle-related genes is highly expansive in HPV+ tumors and dissimilar to HPV− HNSCC [5,67]. These unique gene expression patterns are functionally associated with the significantly higher cell proliferation rate of HPV+ tumor cells compared to HPV− tumor cells. These findings imply that HPV+ HNSCC is a cancer type that is distinct from HPV− HNSCC, and accordingly requires different treatment strategies.

## 3. Differential Immune Responses between HPV+ and HPV− HNSCCs

It is expected that there are fundamental differences in immune responses between HPV+ and HPV− cancers, as HPV+ HNSCC expresses viral proteins as foreign antigens, in addition to other neoantigens created by viral integration and mutagenesis induced by the viral restriction factor APOBEC3 [68,69,70]. In contrast, HPV− HNSCC lacks foreign antigens, rather, they are generated from extensive random mutations or overexpressed cellular genes [16,71]. Accordingly, it is generally accepted that HPV+ HNSCC shows more robust antitumor immune responses compared to HPV− HNSCC. Nevertheless, recent immunotherapy trials have not found any clear benefits of using immune checkpoint inhibitors to treat HPV+ HNSCC patients compared to HPV− HNSCC patients [72,73]. These results suggest that antitumor immune regulations might be extremely complex and cannot be explained as a dichotomic concept. Here, we summarize diverse aspects of immune components and patient outcomes, to better understand the current status, limitations, and future directions.

**Figure 2:**
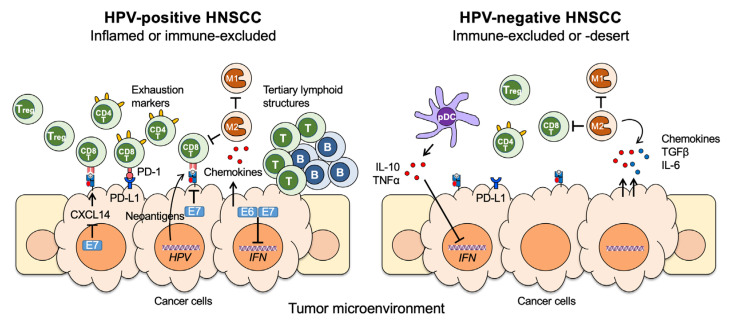
A summary of immune dysregulation and evasion in the tumor microenvironment (TME) of HPV+ (left) and HPV− (right) HNSCCs. The differential immunophenotypes in the TME between HPV+ and HPV− HNSCC are depicted, based on three different spatial distribution of CD8^+^ T cells previously proposed [83,84]. The highly inflamed phenotype of HPV+ OPSCC may be caused by the anatomical distinction of oropharynx composed of the lymphoid tissue. CD4T, CD4^+^ T cell; CD8T, CD8^+^ T cell; Treg, regulatory T cell; B, B cell; T, T cell; M1, M1 macrophage; M2, M2 macrophage; pDC, plasmacytoid dendritic cell; HPV, HPV genome; *IFN*, interferon-related genes; TNFα, tumor necrosis factor-α; TGFβ, transforming growth factor-β; PD-1, programmed death-1; PD-L1, programmed death ligand-1; IL-6, interleukin 6; IL-10, interleukin 10; CXCL14, C-X-C chemokine 14.

As of now, the abundance of tumor infiltrating lymphocytes (TILs) is the most convincing prognostic immune marker in HNSCC, as well as several other cancers [74]. HPV+ HNSCC generally shows higher levels of T cell infiltration, particularly CD8+ T cells, and better clinical outcomes by standard therapy (Figure 2) [75,76,77,78,79]. Over 60% of HPV+ oropharyngeal squamous cell carcinoma (OPSCC) patients show accumulation of stromal or intratumoral CD8+ TIL. Both stromal and intratumoral CD8+ TIL abundance is highly correlated to patient survival [76]. However, the correlation between CD8+ TIL and patient survival seems obscure in HPV− HNSCC [78]. This may be caused by the specific anatomical site that is highly prevalent for HPV+ HNSCC, the oropharynx, where immune cells are relatively abundant. The TME of HPV+ HNSCC also contains higher levels of CD4+ TILs than the HPV− HNSCC TME. While the prognostic value of the CD4+ TILs is still controversial, a subpopulation of CD4+ CD161+ TILs specific to HPV16 positively correlates to patient overall survival [77]. In addition, a recent single cell RNA-seq study of the immune landscape in HPV+ and HPV− HNSCC has revealed that a CD4+ T follicular helper (TFH) cell-related gene expression signature is associated with longer progression-free survival in HNSCC patients [16]. However, it is unclear whether the favorable outcome is due to the antibody reaction mediated by T follicular helper cells, or a mere correlation is caused by the broad enhancement of immune responses [80,81,82].

Interestingly, HPV+ HNSCC shows a significantly higher expression of the immune checkpoint protein PD-1 than HPV− HNSCC (Figure 2). Despite PD-1 playing a key role in inhibiting antitumor effector T cell functions, several studies have shown that the high levels of PD-1 expression in HNSCC is associated with better patient outcome. Indeed, Bhatt et al. have revealed that HPV16-specific T cells are abundant and reactive to almost all viral proteins, E1, E2, E4, E5, and L1, in addition to E6 and E7 [85,86]. These findings suggest that exhausted T cells specific to HPV may indicate previous antitumor immune activation against tumor cells expressing viral antigens, and still maintain certain antitumor potentials. In contrast, the correlation between PD-1 expression and patient outcome is still confusing in HPV− HNSCC [81,87]. Unlike PD-1, high PD-L1 expression on tumor cells correlates to favorable patient survival in HPV− HNSCC, but not in HPV+ HNSCC, while high PD-L1 expression on macrophages is associated with better prognosis [88,89].

Compared to HPV− HNSCC, HPV+ HNSCC also shows a higher expression of Th1 and exhaustion markers, such as CD39, LAG3, PD1, TIGIT, and TIM3 [90]. The expression levels of these T cell exhaustion markers correlate to overall survival of patients with HPV+ HNSCC, but not with HPV− HNSCC [90]. These results imply that reactivation of the exhausted T cells, a significant portion of which might recognize HPV-specific antigens, could be a useful strategy to treat HPV+ HNSCC patients. However, it is still possible that the T cells recognize other tumor-specific antigens generated in HPV+ HNSCC, as a previous study revealed that the majority of tumor-reactive T cells in cervical cancer recognize tumor neoantigens from somatic mutations, or a germline-specific antigen rather than viral antigens [91].

While tumor antigen presentation by major histocompatibility complex I (MHC-I) is critical for antitumor T cell functions, MHC-I expression is controversial. Similar to several viruses, it is well known that HPV has multiple mechanisms through the oncoproteins E5 and E7 to downregulate MHC-I expression on virus-infected host cells (reviewed in [43]). Our previous studies have also shown that expression of all MHC-I α subunits except HLA-F are significantly downregulated in normal keratinocytes containing high-risk HPV in an E7-dependent manner [92,93]. While high-risk HPV E7 is sufficient for the downregulation of HLA-B/C and HLA-E expression, low-risk HPV E7 conversely increases HLA-B/C and HLA-E expression. Further, the downregulation of HLA-C and HLA-E expression were also observed in HPV+ HNSCC, in a comparison using the TCGA RNA-seq data [92]. Wuerdemann et al., also found a loss of MHC-I protein expression more frequently in HPV+ HNSCC than HPV− HNSCC patient samples [89]. In contrast, another study reported that a group of genes in MHC-I and the peptide loading complex are significantly upregulated in HPV+ HNSCC [94]. This discrepancy might be caused by the limitations in the standard criteria of tissue sample collections, spatial and temporal heterogeneity, and appropriate technologies to detect MHC-I expression on cancer cell surface from patient tissues. Thus, further studies using more sophisticated and robust experimental approaches are required for conclusive results.

In addition to T cells, B cell infiltration into the TME is frequently observed and associated with a favorable prognosis [95]. Recent studies have revealed that HPV+ HNSCC has tertiary lymphoid structures (TLS) with germinal center tumor infiltrating B cells (TIL-Bs) and non-organized aggregates containing CD20+ TIL-Bs and CD8+ T cells (Figure 2) [96,97]. Wood et al. also found a B cell-specific gene signature in HPV+ HNSCC, in which the expression is distinct and significantly higher than in HPV− HNSCC [98]. These findings suggest the importance of B cell infiltration into the TME. However, it is still unclear if the TIL-Bs play an important role in antitumor immunity, or just act as bystanders correlated with high levels of TIL.

Tumor-associated M2 macrophages have been suggested as an adverse prognostic factor in HPV− HNSCC, showing that high infiltration of CD163^+^ macrophages is linked to poor patient survival (Figure 2) [99]. However, HPV+ HNSCC does not show this correlation despite a significantly increased infiltration of both M1/M2 and M2 macrophages in the stroma compared to HPV− HNSCC [99]. Infiltration of CD163^+^ type 2 conventional dendritic cells (cDC2) into the TME correlates to a Th1 response and better patient survival in HPV+ HNSCC [100]. In contrast, plasmacytoid dendritic cells (pDCs), known as a protumor DC, significantly suppress IFNα production in HPV− HNSCC via secretion of IL-10 and TNFα in HPV− HNSCC (Figure 2) [101]. Additionally, there is a correlation between pDCs with Tregs in the TME of HPV− HNSCC but not in HPV+ HNSCC [101]. These results suggest that even similar types of macrophages and DCs function differently between HPV+ and HPV− HNSCC patients.

Another interesting difference is the serum levels of the inflammation marker C-reactive protein (CRP), which are significantly higher in HPV+ HNSCC patients compared to HPV− HNSCC. However, high circulating CRP levels are associated with poor overall survival and recurrence-free survival only in HPV− HNSCC patients [102]. HPV+ HNSCC patients have higher serum levels of CRP but better overall survival than HPV− HNSCC patients.

A number of studies have shown the fundamental differences in immune responses between HPV+ and HPV− HNSCC. Nevertheless, recent immunotherapy trials using immune checkpoint inhibitors showed no significant difference between HPV+ and HPV− HNSCC in the response rate [72,103,104]. This is particularly interesting as it is well established that HPV+ HNSCC shows significantly higher expression levels of PD-1 and PD-L1 expression compared to HPV− HNSCC. Thus, it is critical to better understand what other immune factors contribute to antitumor immune responses to clear HPV+ and HPV− HNSCCs differentially.

## 4. Differential Contributions of Oral Microbiome to HPV+ and HPV− HNSCCs

The human microbiome is the ecological system of commensal, symbiotic, and pathogenic microorganisms inhabiting our bodies [105]. Recent studies have shown that changes in microorganism species in the microbiome, despite many of them being commensal, contribute to host immune dysregulation and carcinogenesis [106,107,108,109]. Identification and exploration of the interplay between microorganisms and the human host may provide new insights to a novel treatment axis.

Five bacterial phyla, *Firmicutes*, *Proteobacteria, Bacteroidetes, Fusobacteria,* and *Actinobacteria*, are known to be dominant in the oral microbiota in both healthy individuals and HNSCC patients (Table 1) [110,111]. Nevertheless, significant differences in beta diversity in the microbiome have been observed between healthy buccal mucosal specimens and oral cancer and oropharyngeal tumor tissues [110]. HPV status was also shown to correlate to bacterial abundance. These differences seen when comparing HPV+ and HPV− HNSCC may be due to interactions between the oral microbiota and behavioral/viral risk factors, such as smoking, alcohol, and HPV infection. *Firmicutes* were increased in OPSCC whereas *Bacteroidetes* and *Proteobacteria* were decreased [111]. When looking at genus-level profiles, *Haemophillus* was found to be dominant in HPV− HNSCC samples, while *Veillonella*, *Megasphaera*, and *Anaerolineae* showed a higher abundance in HPV+ HNSCC compared to HPV− HNSCC [111,112]. Interestingly, a longitudinal study showed that HPV+ HNSCC patients who later tested negative for HPV infection post-treatment recorded changes in *Veillonella*, *Lactobacillus*, and *Streptococcus* abundance [111], suggesting a role of HPV related to microbiome expression profiles.

The impact of chronic alcohol consumption in the oral microbiome negatively correlates to the abundance of *Lactobacillus*, one of the most common probiotic microbes [118,119]. The decrease in *Lactobacillus* potentially enhances the alkaline-tolerant bacteria and promotes the growth of ethanol-related pathogens in the oral microbiome, such as *Neisseria* and *Corynebacterium* [120]. *Corynebacterium*, an aerobic, gram-positive genus in the *Actinobacteria* phylum, is associated with the breakdown of toxins found in cigarette smoke and oxidation of ethanol to acetaldehyde (Table 1) [113,121]. The catabolism of tobacco-related toxins slows down carcinogenesis, and accordingly, a higher abundance of *Corynebacterium* is correlated with a lower risk of HNSCC [113].

It is also important to consider that a majority of individuals in the high-risk category due to smoking, alcohol, and/or HPV infection do not develop cancer. One emerging factor to explain this variance is the diverse oral microbiota that differentially affects host immune responses to carcinogenesis. A comparison of the oral microbiome between smokers with and without HNSCC have shown that the smoker HNSCC patients have diminished populations of common commensal bacteria involved in carbohydrate metabolism, such as *Selenomonas, Veillonella*, and *Kingella* (Table 1) [114]. Alternatively, a pathway analysis showed a significant increase in bacterial taxa involved in xenobiotic degradation, such as *Stenotrophomonas*, otherwise absent in healthy individuals [115]. Despite both groups experiencing exposure to the harmful toxins of tobacco, the different oral microbiota is associated with significant different incidence of HNSCC, suggesting that the microbiome plays a role in HNSCC carcinogenesis.

Indeed, the *Fusobacterium* phylum has been discovered to play an important role in promoting HNSCC development independent of tobacco, alcohol, or HPV infection [110]. The bacterial species included in *Fusobacterium* are significantly enriched in HNSCC tissues, causing increased expression of virulence factors in the oral microbiome [110]. Meta-analysis of patient samples also showed a higher abundance of *Fusobacterium* in HNSCC tumor samples compared to both adjacent normal tissues and tissues from healthy individuals (Table 1) [110,116,122,123]. Ironically, however, patients with *F. nucleatum*-positive HNSCC showed a favorable prognosis compared to *F. nucleatum*-negative HNSCC patients [124], while *F. nucleatum* in esophageal cancer is associated with aggressive disease and poor survival [117]. This may be attributed to *F. nucleatum*-mediated modulation of local immunity via downregulation of TNFSF4 and PDGFRβ in fibroblasts, both of which are associated with poor prognosis of HNSCC patients [116]. Additionally, *F. nucleatum* was correlated to low tobacco and alcohol use, suggesting a role in the TME independent of the major risk factors of HNSCC.

Taken together, the oral microbiome plays an important role in HNSCC carcinogenesis. Interactions of the oral microbiota with the major risk factors of tobacco and alcohol can exacerbate their carcinogenic effects. While studies have shown differential microbiota expression associated with HPV infection, additional work is needed to uncover the effect of the altered oral microbiome on HNSCC carcinogenesis. Considering both anatomical location and HPV status, developing novel and combination therapies by targeting the microbiota has great potential to improve patient treatment and outcome.

## 5. Race, Sex, and Disparity in HPV+ and HPV− HNSCCs

There is a clear difference in incidence of HPV+ HNSCC in females compared to males. While the factors that contribute to sexual disparity are largely unknown, several interesting hypotheses have been suggested. As reviewed in Sabatini et al., [125], there may be varying levels of viral exposure to the oropharynx depending on the route of transmission (vaginal-to-oral vs. penile-to-oral). Chatterjee et al. found intriguing differential gene expression profiles in rafts of tonsil, cervical, and foreskin keratinocytes, raising the question of tissue type specific cancer progression [126]. Another consideration is the protective effect of estrogen in cancer development [125]. Beyond differential hormone exposure, there is also the possibility of differential immune responses, such as a more robust antibody-based response occurring with cervical infection in females compared to males [125]. Interestingly, female patients with p16-positive OPSCC showed a higher incidence of HPV+ cervical cancer [127]. Even though the incidence of HPV+ OPSCC is lower in females, the immune response to HPV is important for cancer prevention, and those who are not able to mount an adequate immune response may be more susceptible to future HPV-associated cancers. The importance of the immune response to HPV in the oropharynx is supported by the significantly increased HPV-positive rate in the oropharynx of post renal transplant patients [128].

There is limited prospective clinical trial data regarding sex and racial differences in outcomes for OPSCC. A retrospective study showed a significantly lower survival rate for black patients with HPV− OPSCC, compared to white patients with HPV− OPSCC [129]. This racial disparity has also been observed in HPV+ OPSCC [130,131]. In contrast, a metanalysis by Stein et al. found no evidence of survival disparity in HPV-positive patients by race, while black patients with HPV− OPSCC still show lower survival rates compared to white patients [132]. A multicenter retrospective review by Fakhry et al. found improved survival in females with HPV+ HNSCC in the oropharynx only [133]. These conflicting results among the limited studies may be caused by inconsistent HPV test methods in different studies that detect p16, HPV16 DNA, and/or HPV16 RNA. Indeed, significant variability in HPV testing has been observed in National Cancer Data Base of OPSCC cases, particularly prior to 2015 [134].

To obtain reliable data to determine the degree of sex and race disparities in HNSCC, improved proportionate enrollment of all race and sex groups in prospective clinical trials and/or larger multi-institutional databases with rigorous HPV testing will be required. It will also be important to differentiate clinical outcomes regarding disease mechanisms (immune response, genetic variations, HPV variants, etc.) when comparing factors related to socioeconomic status and demographics.

## 6. Clinical Management of HNSCC: The Role of HPV

### 6.1. Diagnosis, Staging, and Treatment

Despite the complex molecular and immunologic differences between HPV+ and HPV− HNSCC, until recently, treatment approaches for both diseases were uniform. The standard approach for the treatment of HNSCC is multidisciplinary in nature. Diagnosis and staging are commonly initiated by an otolaryngologist with training and experience in HNSCC surgery. Appropriate staging through imaging and endoscopy guides the primary treatment approach. Effective treatment may include single modality or a combination of surgery, radiation, and chemotherapy, depending on the stage of the disease at diagnosis. Tumor size/invasion, location, and the extent of nodal involvement all play a large role in treatment decisions. Staging is a tool to help stratify these treatment decisions, however, is most used as a prognostic tool. Up until recently, HPV status did not play a major role in these guidelines. Recently, the American Joint Committee on Cancer (AJCC) released the eighth edition of the staging guidelines for HNSCC [135]. This guideline has gone through major changes to account for the prognostic significance of HPV status in primary OPSCC. In short, for HPV− OPSCC, no major changes were made, and the staging is comparable to other HPV− HNSCC. However, for HPV+ OPSCC, significant changes were incorporated, allowing for more advanced tumor (T) and nodal (N) stages to be included in overall lower stage groups. These changes are too detailed to encompass in this review, but in short, patients with HPV+ disease can be included in stage I and II categories, even with advanced nodal disease and larger primary tumors. Clinical stage III disease is now limited to those with large, invasive tumors (T4), or bulky (≥6 cm) positive lymph nodes. Pathologic stage III disease includes those with T3/4 primary tumors and N2 (≥5 lymph nodes positive) disease. In a major deviation from other tumor sites, stage IV disease is reserved only for those with metastatic disease. It is important to consider the changes in staging when reviewing older literature, as a direct comparison of stage and outcome for HPV-positive and HPV-negative is not always intuitive.

Incorporating these staging guidelines, treatment for early stage HNSCC often starts with surgical resection followed by observation in some situations. Radiation alone is also an acceptable option for early stage oropharyngeal, hypopharyngeal, and laryngeal tumors [136]. However, these cancers represent the minority of patients, as the most present with advanced disease (positive lymph nodes which do not always correlate to advanced stage with the AJCC eighth edition staging). Multimodality therapy is therefore required for the majority of these patients. For those with advanced stage disease, appropriate radiologic and medical evaluation is needed to determine surgical candidacy. The potential damage to regional tissues, vessels, and organs (i.e., epiglottis, larynx, carotid artery), as well as predicted functional outcome (swallowing/speaking/breathing) must be considered in this decision making. In those with resectable disease, without high risk of organ injury, surgery is often the preferred first approach [136]. In general, adjuvant therapy with radiation therapy is indicated for those with larger primary tumors or those with positive lymph nodes. In patients who undergo surgical resection, but have high risk features, as defined as lymph node extracapsular extension (ECE) or positive margins, adjuvant chemoradiotherapy is recommended based on findings from the MACH-NC meta-analysis [137]. For those who present with surgically unresectable tumors or are at risk of irreversible organ damage with associated poor post-surgical functional outcomes, definitive CRT remains the standard-of-care. CRT is the common approach for organ preservation strategies, particularly in hypopharyngeal and laryngeal tumors, where total laryngectomy is the surgical option. There is some suggestion that regardless of tumor volume, the combination of cisplatin and radiation may be a superior modality for HPV-positive OPSCC; however, this question has not been resolved due to lack of definitive randomized head-to-head trials to evaluate surgery with adjuvant therapy versus CRT. Finally, while the minority of patients present with distant metastatic disease, curative therapies do not exist and palliative treatment with immunotherapy (e.g., PD-1 inhibitors), chemotherapy, and directed radiotherapy are the primary treatment options [136]. 

### 6.2. Surgery

In comparison to the number of well controlled randomized prospective trials for OPSCC treated with CRT, there is a paucity of data for primary surgery as a treatment modality. The stratification based on HPV tests has only been a recent phenomenon in the CRT, as well as surgery clinical trials. Similar to other trials noting improved survival for the HPV+ HNSCC cohort, Heiduschka et al. showed improved survival for the patients both p16-positive and HPV DNA ISH-positive after surgery and adjuvant radiation [138]. Since the p16-negative (mostly HPV−) group has worse survival with CRT, there has been consideration of escalated surgery as an initial modality in HPV− OPSCC. An intriguing study from Spain looking at a mostly HPV− population found a similar survival in HPV+ and HPV− HNSCC cohorts after surgery followed by adjuvant radiation or adjuvant CRT in most of the patients [139]. Sload et al. reviewed the evidence for surgery with adjuvant treatment for HPV− OPSCC [140]. Three studies with limited case numbers showed no difference in survival or locoregional control between HPV+ and HPV− OPSCC treated with surgery followed by adjuvant treatment (reviewed in [140]). Large, randomized trials are needed to further evaluate these findings.

Regarding treatment trends, with the improved survival noted in the HPV+ OPSCC patients with nonsurgical treatment, it is interesting to note a decrease in the number of HPV+ OPSCC treated with surgery from 2010 to 2014 [141]. This may reflect patient preference to avoid surgery when non-surgical options are available, or even could reflect the rapid expansion of de-escalation clinical trials for HPV+ OPSCC over the last decade. Surgery still plays a valuable role in the understanding of OPSCC. Surgery allows for analysis of patient tissue and HPV status beyond what is feasible with nonsurgical treatment. Rubek et al. found that nodal metastasis has 30% lower HPV DNA compared to the primary tumor in a prospective transoral robotic surgery (TORS) cohort [142]. It is possible that HPV DNA loss occurs during migration from the primary tumor site to lymph nodes. However, this finding needs to be confirmed.

One of the arguments for surgery in HPV− OPSCC (especially, TORS) is the potentially improved quality of life in patients with early-stage cancer, by avoiding full dose CRT. However, Michaelsen et al. analyzed the previous studies of quality of life outcomes in OPSCC and found no significant difference between patients with surgical and nonsurgical treatment [143]. The caveat of these studies is the inconsistency of rigorous HPV testing. Unfortunately, an inherent limitation of many studies evaluating surgery for treatment of OPSCC is selection bias of resectable tumors in patients that are healthy enough to tolerate surgery. Despite this limitation, it is clear that outcomes are excellent for carefully selected early-stage HPV+ OPSCC patients treated with surgery [144]. In this single institution series where a significant number of patients refused adjuvant CRT after surgery, locoregional control was 98% and disease specific 5-year survival was 100% [144]. Conclusions regarding superiority or equivalence of treatment will hinge on large clinical trials (ideally randomized), comparing surgery with adjuvant treatment to primary nonsurgical treatment of HPV+ OPSCC.

### 6.3. Multimodality Treatment

As HPV status was recognized as a distinct prognostic group, additional analyses revealed prognosis in HPV+ HNSCC compared to HPV− HNSCC was better, regardless of stage, tobacco use, alcohol use, or treatment strategy [145]. One of the first studies to focus on HPV as a prognostic group was a retrospective analysis of surgically-treated HNSCC by the National Cancer Institute in Milan, Italy (Table 2) [146]. In this cohort of 90 participants, 19% were HPV+ (HPV 16/18 DNA by PCR) and had significantly improved overall and relapse-free survival. Additionally, none developed second primary tumors up to 5 years out of treatment. This led to additional prospective efforts to use HPV status as a correlative biomarker. Eastern Cooperative Oncology Group (ECOG) 2399 was a prospective organ preservation chemoradiation study for resectable stage III or IV oropharyngeal or laryngeal HNSCC, that included a correlative analysis based on HPV status [147]. The study utilized an induction chemotherapy regimen with carboplatin and paclitaxel followed by CRT. In this study, patients with HPV+ HNSCC had an improved response to induction chemotherapy. In addition to an improved response, those with HPV+ disease had improved 2-year overall and progression-free survival [148]. These findings spawned further retrospective and prospective analyses of definitive treatment trials, which uniformly showed improved outcomes in HPV+ disease, as outlined in Table 2.

These outcomes, as well as a large analysis of 1907 HPV+ OPSCC [151], informed the changes made in the AJCC eighth edition staging manual, which separated OPSCC into HPV+ and HPV− diseases [135,152]. Definitive treatment guidelines now dichotomize the treatment approaches for these distinct disease subtypes [136].

### 6.4. Low and Intermediate-Risk HPV+ HNSCC

Further prognostic characterization of HPV+ disease has focused on the definition of low-risk and intermediate-risk disease. Based on clear prognostic differences identified from the Radiation Therapy Oncology Group (RTOG) 0129 study [14], the impact of nodal stage and tobacco use highlighted a distinct survival difference within HPV+ cases. Using a cutoff of greater than 10 pack/year history (PYH) smoking and AJCC seventh edition N2b-N3 (bilateral or >6 cm nodal involvement) for HPV+ HNSCC patients, survival mirrored HPV− HNSCC with less than 10 pack/year smoking history. The 3-year overall survival rate was 70.8% in this intermediate-risk group, contrary to 93.0% in the HPV+ patients with low-risk disease (<10 pack/year history, N0-N2a disease). This intermediate-risk group has been further defined to include those with T4 primary tumors. In a large analysis, by including those with T4 primary tumors and those who have advanced nodal status (N2b-N3) or smoking history of ≥10 pack/year, the HPV+ intermediate-risk group had a higher risk of mortality with an estimated 4-year OS of 68% [153]. Of this group, those with T3N3 or T4N2-N3 disease have 4-year survival rates of 51%, which is equivalent to advanced HPV− disease. Therefore, HPV status alone is not the only factor that contributes to patient outcome. While these tumor and nodal stages have been included in the new AJCC eighth edition staging manual, tobacco use has not. Currently, the proposed intermediate-risk HPV+ disease category is based on AJCC seventh edition stage T1-2N2-N3 or T3-4N0-3 with ≥10 pack/year smoking history OR < 10 pack/year, stage T4N0-N3 or T1-3N2-3. Recent clinical trial efforts focus on treatment strategies for these distinct prognostic disease groups.

### 6.5. Metastatic Disease: Do *HPV+* HNSCC Patients Fare Better?

While prognosis of HPV+ HNSCC is generally better than HPV− HNSCC, approximately 10% of patients still develop R/M disease [14,148,149,154]. Those with HPV+ HNSCC also have unique metastatic patterns, often with multiple organs involved and atypical sites, such as the bone and liver [155]. Additionally, the median time to development of distant metastases following curative treatment is longer than HPV− HNSCC, with one study demonstrating 16.4 vs. 7.2 months (*p* = 0.008) [156]. Furthermore, metastatic disease development after 5 years in HPV+ HNSCC patients has been described [154,157]. Despite these findings, those with HPV+ HNSCC and relapse following definitive therapy still have improved prognosis in terms of survival over their HPV− HNSCC. In one analysis, 2-year overall survival after relapse was 54.6% in HPV+ HNSCC vs. 27.6% in HPV− HNSCC [158]. This improved survival in part has been suspected to be related to more responsive disease. However, in a retrospective analysis of the landmark EXTREME trial evaluating platinum, fluorouracil, and the addition of cetuximab in R/M HNSCC, survival outcomes were better in both groups by adding cetuximab [159]. A sub-analysis demonstrated that HPV+ HNSCC did have improved survival in the R/M setting over their HPV− counterparts, however this was not statistically significant (12.6 vs. 9.7 months, *p* = 0.092) [159]. The analysis is limited, however, as only 10% of evaluable participants had p16+ disease.

### 6.6. Immunotherapy Trials: Impact of *HPV* Status

As monoclonal antibody inhibitors of the PD-1 and PD-L1 interaction have emerged as the preferred treatment option for R/M HNSCC, sub-analyses of outcomes based on HPV status have been performed. In the phase IB KEYNOTE-012 study evaluating the pembrolizumab monotherapy in an R/M HNSCC cohort, 23% of participants were HPV+ based on p16 status [103]. Response rates were higher in those with HPV+ HNSCC (ORR 24% vs. 16%) [160]. However, in the larger, phase II KEYNOTE-055 study evaluating pembrolizumab monotherapy in R/M HNSCC after progression on platinum therapy, ORRs were similar in those with HPV+ (16%, 95% CI 6–32) and HPV− disease (15%, 95% CI 10–23) [104]. The landmark phase III CHECKMATE-141 study compared the PD-1 inhibitor, nivolumab, to second-line chemotherapies in R/M HNSCC. Of those tested and successfully treated with nivolumab, 26.4% of participants had HPV+ HNSCC based on p16 status and 20.8% HPV− HNSCC. While the aggregate study population had an improved OS with nivolumab treatment in this study, a post hoc analysis revealed that this survival benefit was more profound in HPV+ HNSCC patients (Supplementary Figure S4 in [72]). Interestingly, expression of PD-L1 (≥1% expression by IHC) was a stronger biomarker for this survival benefit, however even those with HPV+ PD-L1 negative disease had improved OS with nivolumab compared to chemotherapy (OS 10 vs. 6.4 months, HR 0.55 95% CI 0.22–1.39). This difference was not seen in HPV− PD-L1 expressing HNSCC (OS 7.1 vs. 7.4 months HR 0.82 95% CI 0.82 (0.31–2.19)) [161]. A subsequent study with a neoadjuvant PD-1 inhibitor arm, CHECKMATE 358, evaluated neoadjuvant nivolumab for previously untreated, locally advanced HNSCC. In this study, the pathologic response to therapy was higher in the HPV+ HNSCC group (*n* = 4/17; 23.5%) than the HPV− group (*n* = 1/17; 5.9%) [162]. Taking these findings together, HPV+ HNSCC patients have shown marginal signal for increased clinical benefit from PD-1 blockade treatment over those with HPV− disease.

Based on these findings, evaluation of PD-L1 expression based on HPV status has been an area of interest. Early work by Lyford-Pike et al. demonstrated localized PD-L1 expression in the tonsillar reticulated epithelium of the deep crypts, which represent the site of HPV-associated carcinogenesis [163]. Additionally, the authors demonstrated higher PD-L1 staining by immunohistochemistry (≥5% cells positive) in HPV+ HNSCC (14/20; 70% positive) compared to HPV− HNSCC (2/7; 29% positive) [163]. Larger studies have shown a higher expression in HPV+ HNSCC than HPV− HNSCC [164,165,166], while no other marker shows a significant difference in expression by HPV status [167,168]. These findings have been criticized, however, as there has been significant heterogeneity in scoring systems and assays utilized [169]. Regardless, it is apparent that a benefit from PD-1 blockade is seen in both HPV+ and HPV− HNSCCs. While PD-L1 expression shows some benefits in predicting the treatment response, it is not definitive. PD-L1 inhibitors emerged as the preferred standard-of-care for many patients with R/M HNSCC, and are moving into earlier stage disease. Thus, novel biomarkers are being investigated to enrich treatment strategies.

## 7. Current Treatment Approaches for HPV+ and HPV− HNSCC

### 7.1. De-Intensification or Intensification: Risk Adapted Therapy

While multimodality therapy is often utilized across the anatomic sites of HNSCC, the management of OPSCC has become increasingly complex as our understanding of HPV status has grown. Patients with HPV+ OPSCC often present with advanced nodal disease at diagnosis and are not candidates for surgical resection, either due to having unresectable disease or a high risk of morbidity. Surgical innovations including TORS have emerged as a strategy to minimize the morbidity through use of natural orifice access approaches to the surgical site. TORS has been shown to optimize post-operative functional outcomes for carefully selected patients compared to traditional open approaches. However, large volume tumors or loss of important anatomic structures would still result in poor functional outcomes, regardless of the approach to the cancer. In addition, many TORS patients require adjuvant radiation or CRT as part of their treatment. Furthermore, as our understanding of the prognostic significance of HPV has grown, CRT approaches are evolving. Currently, the concept of de-intensification and intensification have emerged for HNSCC.

As previously discussed, in the clinical trial setting, patients presenting with locally advanced HPV+ HNSCC are being dichotomized into low-risk and intermediate-risk groups. In addition to staging, smoking status has a large impact on these risk groups. For those with low-risk disease, extensive efforts are being made to pursue de-intensification therapy. As the long-term cure rates exceed 90% in this risk group with standard treatment, there is strong support to consider reducing the intensity of treatment for this group to spare them from long-term toxicity. While this is not yet the standard-of-care, numerous clinical trials have been completed and are underway to determine the best strategy. Pivotal, randomized clinical trials that have been completed and that are actively accruing are outlined in Table 3. In summary, different approaches in surgical, post-operative radiation therapy (PORT), induction chemotherapy, addition of immunotherapy, and novel types of chemoradiotherapy are being evaluated. Thus far, completed phase III trials have not identified a clear strategy for de-escalation. Ongoing efforts seek to decrease radiation dose both with PORT and definitive therapy, as well as incorporate PD-1 blockades and novel chemotherapy regimens into treatment. Many centers are adopting these as part of their treatment approach through these clinical trials.

For those who present with intermediate-risk HPV+ HNSCC, treatment approaches are often the same as for those with HPV− HNSCC. For resectable patients, adjuvant RT at standard dose (typically 60 Gy/2 fractions per day over 6 weeks) is generally pursued unless there is very early-stage disease [136]. For those with the high-risk features of extranodal extension (ENE) or positive margins, or multiple intermediate risk features (lymphovascular invasion or LVI, perineural invasion or PNI (>3 positive lymph nodes), adjuvant cisplatin-based CRT is the standard [136]. Many of those with HPV+ intermediate-risk HNSCC present with advanced T-stage (T4) and N-stage (N2-3), and are not candidates for surgical resection. Consequently, cisplatin-based CRT (70 Gy/2 fractions per day over 7 weeks) is often the mainstay of treatment for this group as a definitive therapy. As the cure rates for this population remain stagnant and inferior to low-risk HPV+ HNSCC, treatment intensification clinical trials have emerged as the investigational approach. As those with HPV+ intermediate-risk HNSCC have comparable prognosis to patients presenting with HPV− HNSCC, these groups are often included in these trials together. Table 4 outlines past and current treatment intensification approaches.

In summary, prior approaches have included accelerated fraction radiation, induction chemotherapy, and chemotherapy combinations with radiation. However, none of these have led to a deviation from the current standard of cisplatin-based CRT. With the activity of PD-1 inhibitors in R/M disease, there is excitement about their impact in the definitive setting. Initial studies demonstrated safety and early favorable efficacy signal [180]. However, one large, randomized trial of the PD-L1 inhibitor, avelumab, has already read out as negative, thus dampening the enthusiasm for these agents in this treatment setting [179]. While these unique treatment approaches are rapidly moving forward, the standard-of-care for curative intent therapy has remained the same for decades regardless of HPV status. While those with low-risk HPV+ disease enjoy high survival rates, some still will recur. For those with intermediate-risk HPV+ disease and HPV− disease, relapse rates are generally higher. Those that recur or who present with metastatic disease at diagnosis have incurable disease. However, recent advances in therapies seek to improve outcome for these patients as well.

### 7.2. Advances in the Treatment of Recurrent/Metastatic Disease: Implications of HPV Status

Approximately 10–15% of patients with HNSCC will develop R/M disease either at diagnosis or following treatment [181,182]. For these patients, systemic therapy remains the mainstay for disease control and optimization, quality of life, and survival. As mentioned above, PD-1 inhibitors have become the standard-of-care for most patients, either as monotherapy or in combination with chemotherapy. Despite there being no clear link between HPV status and response to these agents, significant efforts are being made to look at novel immunotherapy approaches to treat HPV+ HNSCC. Much of this has been led by the emergence of therapeutic vaccines. As HPV-infected cancer cells express viral oncoproteins, these represent antigenic targets for vaccine development. Several vaccines using different technologies have emerged. A number of viral vector- [183], DNA- [184], and peptide- [68] based therapeutic vaccines are in various stages of development. Due to the immunologic nature of response, combination with other immunotherapies is being pursued, including combinations with PD-1/PD-L1 inhibitors [185]. Taking this a step further, with the rapid growth of cell-based therapies in hematologic malignancies, HPV-specific cell-based therapies are gaining traction in HPV+ HNSCC. HPV16/18 E6/E7 adoptive T cell therapies are currently in early-phase clinical trials in HNSCC, and seek to bring cell-based therapies into the treatment of this disease (NCT03578406, NCT02379520) [186]. Other cell-based therapies, including one that targets melanoma-associated antigen 4 (MAGEA4), seek to expand cell-based therapy beyond HPV+ HNSCC (NCT04408898). Development of novel immunotherapies is rapidly moving forward, and in combination with these HPV-specific therapies is certain to be an area of future research.

Beyond immunotherapies, targeted therapies are another area of growing research in HNSCC. As discussed above, a number of different pathways are altered in HNSCC. Epidermal growth factor receptor (EGFR/ERBB1) and its family ERBB2 (HER2), ERBB3 (HER3), as well as FGFR kinase aberrations have been demonstrated in HNSCC (Figure 1). Cetuximab, a monoclonal antibody inhibitor of EGFR, has been an approved treatment both in combination with radiotherapy for definitive treatment and in the metastatic setting. Until recently, this agent was often utilized as a standard chemosensitizing agent with definitive chemoradiotherapy. However, recent findings from the De-ESCALaTE HPV, RTOG 1016, and TROG 12.01 trials (Table 3) in HPV+ HNSCC showed inferior results with cetuximab versus cisplatin as a CRT in the definitive setting. While this agent is still utilized in the R/M setting in HPV+ HNSCC, interest in the definitive setting has waned. Despite this, a pan-ERBB inhibitor, Aafatinib, achieved NCCN support for use for R/M HNSCC as second-line therapy following platinum failure, based on the LUX-HN study [145,187]. Beyond these receptor tyrosine kinases, potentially druggable alterations in other common downstream kinases including MAPK (*HRAS, NRAS, KRAS, BRAF, NF1*) and *PI3K/AKT/mTOR* pathway have been seen in HNSCC [188]. Of these, the *PIK3CA* mutation has been more commonly seen in HPV+ HNSCC [189], while *HRAS* mutations are more common in HPV− HNSCC [190]. Targeted therapies for each of these are being pursued, however development is not intentionally exclusive to HPV status [191,192]. Additional interest in the cell cycle pathway (*CDK4/6, CCND1, CDKN2A*) and DNA repair pathway (*BRCA, ATM, PALB2)* is growing, with trials underway in HNSCC as a whole [193,194,195]. As the molecular characteristics of HPV+ and HPV− HNSCCs are understood, it is surmised that disease-specific treatments will emerge.

## 8. Conclusions

As discussed above, there have been significant advances in our understanding of molecular mechanisms and improvement of clinical approaches in HNSCC. Nevertheless, there are more questions than answers from current research, as we currently do not have clear explanations on several important issues in HNSCC. First, it is still mysterious how HPV+ HNSCC patients show substantially better survival and clinical outcomes after standard-of-care treatment than HPV− HNSCC patients. Studies by ourselves and others have suggested potential factors, such as high cell proliferation and DNA damage rates of HPV+ cancer cells compared to HPV− cancer cells [5,196]. In contrast, several studies have suggested that HPV+ HNSCC in anatomical sites other than the oropharynx and tonsil does not show favorable outcomes [197,198,199]. However, as positive p16 immunohistochemistry for non-oropharyngeal head and neck cancers does not accurately represent HPV status [200,201], further molecular analyses and mechanistic studies would be necessary for any definitive answer. Second, the extreme bias of HNSCC prevalence in the male population is obvious but still unexplainable. Recent studies have discovered that females have more robust immune responses against various viruses, including HPV and SARS-CoV-2 [202,203]. The sex differences in immune responses and cancer development are suspected to be caused by sex hormones and differential behaviors between males and females [204,205]. Third, while expression levels of PD-1 and PD-L1 in HPV+ HNSCC are undoubtedly higher compared to HPV− HNSCC, there is no evident benefit from immunotherapy using PD-1/PD-L1 inhibitors. In addition, there is an urgent need to develop useful biomarkers available to identify the intermediate-risk group of HPV+ HNSCC and responders from non-responders to current therapies. A better understanding of microbiota linked to HNSCC would also be helpful, as the oral regions form an abundant and diverse microbial ecosystem that may contribute to immune dysregulation and carcinogenesis. Considering the extraordinary heterogeneity in HNSCC developed in the complex and dynamic anatomical sites, cancer cell evolution in interaction with proximal and distal organs could also be a new area to explore. Taken together, basic, translational, and clinical research in HNSCC during the next decade will bring about scientific breakthrough, as well as innovative treatment for cancer patients.

## Figures and Tables

**Table 1:** Microbiome studies in HNSCC patients.

Type	Samples	Population	Major Findings
Longitudinal Cohort Study	59 (from 42 patients)	17 HNSCC (7 HPV+ OPSCC, 4 HPV− OPSCC, 6 HPV− OSCC), 25 control	Shift in bacterial abundance in HPV+ OPSCC following treatment; microbial diversity may be used as a diagnostic for HNSCC [111]
	100 (from 50 patients)	Tumor and non-tumor sites from OSCC patients	Increased richness and diversity in OSCC tumor sites; higher prevalence of *Prevotellaceae, Fusobacteriaceae, Flavobacteriaceae, Lachnospiraceae, Peptostreptococcaceae*, and *Campylobacteraceae* in OSCC [110]
	83	Tumor and anatomically matched normal tissue from oral cancer and pre-cancer	Reduction of *Firmicutes* and *Actinobacteria* in cancer [112]
Prospective study	383	129 HNSCC and 254 matched controls	*Corynebacterium* and *Kingella* are associated with a lower risk of HNSCC [113]
	38	18 oral cavity squamous cell cancer (OCSCC), 8 pre-malignant lesions, 12 control	*Fusobacterium, Prevotella, Alloprevotella*-enriched in OCSCC [114]
	51	27 smokers with and 24 without HNSCC	Higher relative abundance of bacteria involved in xenobiotic and amine degradation in HNSCC [115]
Retrospective analysis	151	Oral squamous cell carcinoma (OSCC) patients	*F. nucleatum*-associated OSCC is associated with favorable prognosis [116]
	325	Esophageal Cancer (300 SCC, 12 adenocarcinomas, 13 others)	*F. nucleatum* is a potential biomarker for esophageal cancer is associated with poor prognosis [117]

**Table 2:** Key multimodality treatment trials demonstrating improved outcome in HPV+ HNSCC.

Source	Parent Study	Years	Treatment	Design	Number of Patients in Analysis	Disease Sites	HPV Assessment	% HPV+/HPV−	Outcome (HPV+ vs. HPV−)
Licitra, et al., 2006 [146]	NA	1990–1999	Surgery followed by RT	Single-arm, retrospective	90	OP	HPV 16/18 DNA PCR	19/81	5-year OS (79% vs. 46%), 5-year incidence tumor relapse (21% vs. 53%), 5-year incidence second primary (0 vs. 12%)
Fakhry, et al., 2008 [148]	E2399	2001–2004	IC followed by CRT	Single-arm Phase II	96	OP, Larynx	HPV types 16, 33, 35 DNA ISH	39.6/60.4	ORR to IC (82% vs. 55%, *p* = 0.01) and CRT (84% vs. 57% *p* = 0.007, 2-year OS (95% vs 62%, *p* = 0.005),
Ang, et al., 2010 [14]	RTOG 0129	2002–2005	CRT (accelerated fx vs. standard RT)	Randomized phase III	323	OP	HPV 16, 18, 31, 33, 35, 39, 45, 51, 52, 56, 58, 59, and 68 DNA ISH	63.8/36.2	3-year OS (82.4%, vs. 57.1%, *p* < 0.001), 3-year PFS (73.7% vs. 43.4%, *p* < 0.001)
Rischin, et al., 2010 [149]	TROG 02.02	2002–2005	CRT	Randomized phase III	182	OP	P16 IHC	57.3/42.7	2-year OS (91% vs. 74%, *p* = 0.004), 2-year FFS (87% vs. 72%, *p* = 0.003)
Posner, et al., 2011 [150]	TAX 324	1999–2003	IC followed by CRT	Randomized Phase III	111	OP	HPV E6/E7 PCR	50/50	* OS (79% vs. 31%, *p* < 0.0001, PFS (73% vs. 29%, *p* < 0.0001), LRF (13% vs. 42%, *p* = 0.0006)

* analysis was at 83 months for HPV+ and 82 months for HPV−.

**Table 3:** Key de-intensification trials in HPV+ HNSCC.

Trial	Design (No. of Patients)	Patient Population	De-Escalation Strategy and Regimens (* De-Escalation Arm)	Primary Outcome Measure	Status	Summary of Findings (if Completed)
RTOG 1016 [170]	Randomized, noninferiority phase 3 (*n* = 987)	AJCC 7th ed. T1-T2, N2-3 or T3-T4, N0-N3; any smoking history	Chemotherapy * Cetuximab 400 mg/m^2^ then 250 mg/m^2^ q1w × 7 + RT (70 Gy/35 fx in 6 weeks) Cisplatin (100 mg/m^2^) 3w × 2 + RT (70 Gy/35 fx in 6 weeks)	OS	Completed	Cetuximab was not shown to be non-inferior to cisplatin
De-ESCALaTe [171]	Randomized phase 3 (*n* = 334)	AJCC 7th ed. T3-T4, N0 or T1-T4, N1-N3; <10 PYH	Chemotherapy * Cetuximab 400 mg/m^2^ then 250 mg/m^2^ q1w 7 + RT (70 Gy/35 fx in 6 weeks) Cisplatin (100 mg/m^2^) 3w × 3 + RT (70 Gy/35 fx in 6 weeks)	OS and late toxicity	Completed	Mean number of severe events and all grade toxicity the same in both groups
TROG 12.01 [172]	Randomized phase 3 (*n* = 189)	AJCC 7th ed. Stage III (excluding T1-2N1) or stage IVA-B (excluding T4 and/or N3 and/or N2b-c); ≤10 PYH of smoking	Chemotherapy * Cetuximab 400 mg/m^2^ then 250 mg/m^2^ q1w × 7 + RT (70 Gy/35 fx in 6 weeks) Cisplatin (40 mg/m^2^) 1w × 7 + RT (70 Gy/35 fx in 6 weeks)	Difference in AUC of MDADI from baseline to 13 weeks post-therapy	Completed	No difference in AUC of MDADI between groups; Worse 3-year FFS in cetuximab (80% [95% CI: 70–87%]) vs. cisplatin (93% [95% CI: 86–97%])
ORATOR [173]	Randomized Phase II (*n* = 68)	AJCC 7th ed. T1-T2, N0-2 (≤4 cm); any smoking history	Surgery * TORS and neck dissection+/− adjuvant RT or CRT (based on pathology) CRT (RT 70 Gy, various chemo regimens)	1-year swallowing QoL by MDADI	Completed	1-year MDADI score higher in RT group
ECOG 3311 [174]	Randomized phase II (*n* = 519 overall, 209 Arms B and C)	AJCC 7th ed. stage III-IVA resected, intermediate pathologic risk (close margins [<3 mm], 2–4+ nodes or 1 node >3 cm and ≤6 cm, ENE ≤ 1 mm, or PNI/LVI); any smoking history	PORT * 50 Gy PORT Arm B 60 Gy PORT Arm C	2-year PFS	Completed	2-year PFS similar in Arm B 95.0% (90% CI = 91.4%, 98.6%) to ARM C 95.9% (90% CI = 92.6%, 99.3%)
NRG-HN002 [175]	randomized, phase II trial (*n* = 306)	AJCC 7th ed. T1-T2 N1-N2b M0, or T3 N0-N2b M0; ≤10 PYH of smoking	No chemotherapy * 60 Gy IMRT over 5 weeks 60 Gy IMRT over 6 weeks + cisplatin 40 mg/m^2^ Q1w × 6	2-year PFS and 1-year swallowing QoL by MDADI	Completed	Similar 2-year PFS (88% RT vs. 91% CRT), but RT alone did not meet pre-specified 2-year PFS goal
PATHOS [NCT02215265]	Randomized phase II/II (*n* = up to 1100)	AJCC 7th ed. T1-T3, N0-N2b 8th edition stage T1-T3, N0-N1; Any smoking history (except current smokers with N2b disease)	PORT Arm B1: PORT 60 Gy over 6 weeks * Arm B2: PORT 50 Gy over 5 weeks Arm C1: POCRT 60 Gy over 6 weeks with Cisplatin (high risk features) * Arm C2: PORT 60 Gy over 6 weeks without chemotherapy (high risk features)	1-year MDADI and Overall survival	Ongoing	NA
NRG-HN005 [NCT03952585]	Randomized phase II/III (*n* = up to 711)	AJCC 8th ed. 8th T1-2N1M0 or T3N0-N1M0; ≤ 10 PYH of smoking	Radiation and chemotherapy Arm 1: RT 70 Gy over 6 weeks + Cisplatin 100 mg/m^2^ Q3w × 2 * Arm 2: RT 60 Gy radiation over 3 weeks + Cisplatin 100 mg/m^2^ Q3w × 2 * Arm 3: RT 60 Gy over 3 weeks + Nivolumab 240 mg Q2w × 6	Phase II, PFSPhase III, PFSand QoL by the MDADI global score	Ongoing	NA
DART-HPV [NCT02908477]	Randomized phase III (*n* = 227)	TORS resected primary disease with either AJCC 8th ed. T3/4 or N2b disease, and/or ENE, LVI, PNI	POCRT * RT 30 Gy/1.5 Gy fractions BID (intermediate risk) or 36 Gy/1.8 Gy BID fractions (high risk) + Docetaxel 15 mg/m^2^ days 1, 8 RT 60 Gy/2 Gy fractions daily alone (intermediate risk) or with cisplatin 40 mg/m^2^ Q1w × 6 (high risk)	Adverse event rate (late grade 3–5 toxicities)	Ongoing	NA

* De-Escalation Arm.

**Table 4:** Selected treatment intensification trials.

Trial	Design (No. of Patients)	Patient Population	Intensification Strategy and Regimens (* Intensification Arm)	Primary Outcome Measure	Status	Summary of Findings (if Completed)
RTOG 0129 [176]	Randomized Phase III (*n* = 721)	AJCC 6th ed. Stage III-IVB OC, OP, HP, Larynx; any HPV risk group	Accelerated fraction (AFX) RT * AFX: 72 Gy in 42 fx over 6 weeks + Cisplatin 100 mg/m^2^ Q3W × 2 SFX RT 70 Gy in 35 fx over 7 weeks + Cisplatin 100 mg/m^2^ Q3W × 3	OS	Completed	No difference in OS (HR, 0.96; 95% CI, 0.79 to 1.18; *p* = 0.37).
RTOG 0522 [177]	Randomized Phase III (*n* = 891)	AJCC 6th ed. stage III-IVB OC, OP, HP, Larynx; any HPV risk group	Combination chemotherapy Arm 1: AFX RT + Cisplatin 100 mg/m^2^ Q3W × 2 * Arm 2: AFX RT + Cisplatin 100 mg/m^2^ Q3W × 2 + Cetuximab 400 mg/m^2^ then 250 mg/m^2^ q1w × 7	PFS	Completed	No difference in PFS, OS, LRF. Higher acute toxicities with the addition of cetuximab
PARADIGM [178]	Randomized Phase III (*n* = 145)	AJCC 6th ed. Stage IVA-IVB (T3/T4 or N2/N3, but not T1/N2) OC, OP, Larynx; any HPV risk group	Induction chemotherapy * Arm 1: Docetaxel 75 mg/m^2^, Cisplatin 80 mg/m^2^, 5-FU 800 mg/m^2^/d days 1–4 Q3w × 3 followed by CRT with carboplatin or docetaxel Arm 2: AFX RT + cisplatin 100 mg/m^2^ Q4W × 2	OS	Completed	No difference in OS (HR, 1.09; 95% CI 0.59–2.03). poor accrual (145 of 300 planned)
JAVELIN-Head and Neck 100 [179]	Randomized Phase III (*n* = 697)	AJCC 7th ed. HPV- Stage III, IVA, IVb disease; non-OP HPV+Stage III, IVA, IVB disease; HPV+ OP T4 or N2c or N3 disease	Combination PD-1 blockade + CRT * Avelumab SFX RT 70 Gy over 7 weeks SFX RT 70 Gy in 35 fx over 7 weeks + Cisplatin 100 mg/m^2^ Q3W × 3	PFS	Completed	Median PFS was not reached in either group, however stratified hazard ratio (1.21 [95% CI 0.93–1.57]) favored the placebo group (one-sided *p* = 0.92)
KEYNOTE-412 [NCT03040999]	Randomized Phase III (*n* = 780 planned)	AJCC 7th ed OP HPV+ (any T4 or N3), OP HPV− (any T3-T4 or N2a-N3), or larynx/HP/OC (any T3-T4 or N2a-N3)	Combination PD-1 blockade + CRT * Pembrolizumab + CRT (AFX or SFX 70 Gy) + Cisplatin 100 mg/m^2^ Q3W × 2–3 CRT (AFX or SFX 70 Gy) + Cisplatin 100 mg/m^2^ Q3W × 2–3	Event Free Survival (EFS)	Ongoing	NA
KEYNOTE-689 [NCT03765918]	Randomized Phase III (*n* = 704 planned)	AJCC 8th ed. resectable, stage III/IVA HPV− or T4N0-2 HPV+	Neoadjuvant PD-1 blockade * Pembrolizumab Q3W × 2 doses pre-operatively followed by risk-adapted PORT or POCRT (cisplatin) + pembrolizumab Surgery followed by adjuvant risk adapted PORT or POCRT (cisplatin)	Major Pathological Response (mPR) and EFS	Ongoing	NA
RTOG 1216 [NCT01810913]	Randomized phase II/III (*n* = 480 planned)	Resected AJCC 7th ed Stage III-IVB HPV+ or HPV− disease with high-risk features (ENE or positive margins)	Adjuvant POCRT + PD-L1 blockade POCRT 60 Gy + cisplatin 40 mg/m^2^ Q1W × 6 POCRT 60 Gy + docetaxel 15 mg/m^2^ Q1w × 6 + cetuximab + Cetuximab 400 mg/m^2^ then 250 mg/m^2^ q1w × 7 POCRT 60 Gy + cisplatin 40 mg/m^2^ Q1W × 6 + Atezolizumab 1200 mg Q3W × 8	Phase II-DFSPhase III-OS	Ongoing	NA

* Intensification Arm

## Abbreviations

RT	radiation therapy
OP	oropharynx
RFS	relapse-free survival
OS	overall survival
PFS	progression-free survival
ORR	overall response rate
LRF	locoregional failure
IC	induction chemotherapy
CRT	chemoradiotherapy
ISH	in situ hybridization
IHC	immunohistochemistry
Fx	fractions
Gy	Gray
BID	twice a day
AUC	area under the curve
MDADI	MD Anderson Dysphagia Inventory
PORT	post-operative radiation therapy
POCRT	post-operative chemoradiotherapy
IMRT	intensity modulated radiation therapy
QoL	quality of life
SFX	standard fraction
